# The iron chelator Deferasirox causes severe mitochondrial swelling without depolarization due to a specific effect on inner membrane permeability

**DOI:** 10.1038/s41598-020-58386-9

**Published:** 2020-01-31

**Authors:** Esther M. Gottwald, Claus D. Schuh, Patrick Drücker, Dominik Haenni, Adam Pearson, Susan Ghazi, Milica Bugarski, Marcello Polesel, Michael Duss, Ehud M. Landau, Andres Kaech, Urs Ziegler, Anne K. M. Lundby, Carsten Lundby, Petra S. Dittrich, Andrew M. Hall

**Affiliations:** 1 Institute of Anatomy, University of Zurich, Zurich, Switzerland; 2 Department of Biosystems Science and Engineering, ETH Zurich, Zurich, Switzerland; 3 Center for Microscopy and Image Analysis, University of Zurich, Zurich, Switzerland; 4 Department of Chemistry, University of Zurich, Zurich, Switzerland; 5 Institute of Physiology, University of Zurich, Zurich, Switzerland; 6 Department of Nephrology, University Hospital Zurich, Zurich, Switzerland

**Keywords:** Kidney, Nephrology

## Abstract

The iron chelator Deferasirox (DFX) causes severe toxicity in patients for reasons that were previously unexplained. Here, using the kidney as a clinically relevant *in vivo* model for toxicity together with a broad range of experimental techniques, including live cell imaging and *in vitro* biophysical models, we show that DFX causes partial uncoupling and dramatic swelling of mitochondria, but without depolarization or opening of the mitochondrial permeability transition pore. This effect is explained by an increase in inner mitochondrial membrane (IMM) permeability to protons, but not small molecules. The movement of water into mitochondria is prevented by altering intracellular osmotic gradients. Other clinically used iron chelators do not produce mitochondrial swelling. Thus, DFX causes organ toxicity due to an off-target effect on the IMM, which has major adverse consequences for mitochondrial volume regulation.

## Introduction

Mitochondria are complex organelles with a variety of functions^1^. In most cell types, they have a characteristic elongated shape and form an inter-connected network^2^. Mitochondrial morphology is highly dynamic and intricately related to function^3^. Although much has been learnt in recent years concerning the molecular mechanisms underlying mitochondrial fission and fusion, the key factors that regulate mitochondrial volume and shape within cells remain less clear^4^. Pathological mitochondrial swelling occurs in various critical diseases, such as ischemia-reperfusion injury, and the majority of previous studies have attributed this to opening of a large non-specific pore in the IMM called the mitochondrial permeability transition pore (mPTP) (e.g. see^5–9^). Opening of the mPTP causes immediate depolarization of mitochondria and the release of mitochondrial proteins like cytochrome c. However, the identity and nature of the mPTP remain disputed^10–12^, and clinical trials of mPTP inhibitors have been disappointing^13^. There is, therefore, a need to consider other mechanisms that might lead to mitochondrial swelling in certain pathological states.

Toxicity from therapeutic drugs is a major cause of organ dysfunction in humans^14^. Mitochondria are frequently targets of toxicity in aerobic organs such as the heart, kidney and liver^15^. It is often tacitly assumed that toxicity occurs due to inhibition of respiratory chain (RC) function and/or induction of oxidative stress; however, there may be other ways by which drugs can harm mitochondria. Increasing understanding of how drugs affect mitochondria not only facilitates rational redesign to reduce toxicity, but also provides the potential to gain important new insights into basic mitochondrial biology.

Deferasirox (DFX, marketed as Exjade) is an iron chelator widely used in patients at risk of the effects of iron overload (e.g. due to recurrent blood transfusions)^16^. It is also currently being investigated as a novel therapy for cancer^17^. DFX belongs to the N-substituted bis-hydroxyphenyl-triazole class of tridentate iron chelators, and was approved for usage by the FDA in 2005^16^. Unlike older chelators it can be taken orally, which is much more convenient for patients. Although initial studies in humans suggested a generally good safety profile, numerous cases of significant organ toxicity have since been reported^18–25^, along with suspected deaths, leading to boxed warnings from the FDA^26^. While toxicity can occur in various organs, including the gastro-intestinal tract and bone marrow, the kidney and liver are most frequently affected since these are the major excretion pathways for the drug^26^. Renal toxicity occurs in over 20% of patients and is localized to the proximal tubule (PT)^26^, which has a very high density of mitochondria and is dependent on aerobic metabolism to generate ATP^27^.

Previous *in vitro* studies have suggested that DFX induces apoptosis in PT cells, possibly via a deleterious effect on mitochondria^28,29^. Iron is essential for various aspects of mitochondrial function; for example, iron-sulphur clusters are co-factors in components of the RC, the citric acid cycle and anti-oxidant defenses^30^. Thus, depletion of mitochondrial iron by DFX might lead to adverse changes in RC activity or redox state, which could explain the observed toxicity^26^. However, other clinically used iron chelators are not associated with kidney disease. Moreover, iron chelators can also have beneficial effects in mitochondria; for example, by inhibiting cell death due to ferroptosis^31^. Therefore, the reason why DFX alone causes toxicity in organs like the kidney has remained a conundrum.

Here, using a variety of methods, we show that DFX is indeed a potent mitochondrial toxin. However, rather than causing RC inhibition, oxidative stress or mitochondrial fragmentation, it instead induces severe swelling of these organelles. Other clinically used iron chelators do not produce the same effect, thus explaining why DFX is more toxic in humans. Remarkably, we observed that mitochondria exposed to DFX remain energized, even when grossly swollen, which prompted us to look for a mechanism other than mPTP opening. We subsequently found that DFX has a direct, but subtle effect on the permeability of the IMM, which results in an influx of water into the matrix and partial uncoupling of the RC, but without causing depolarization. Moreover, we provide evidence that DFX-induced swelling can be prevented by manipulating intracellular osmotic gradients across the IMM. In summary, in addition to revealing a previously unknown disease mechanism, our findings suggest that the movement of water across the IMM plays a critical role in the regulation of mitochondrial morphology within living cells and the genesis of pathological swelling.

## Results

### Deferasirox causes acute mitochondrial swelling without depolarization

**Figure 1:**
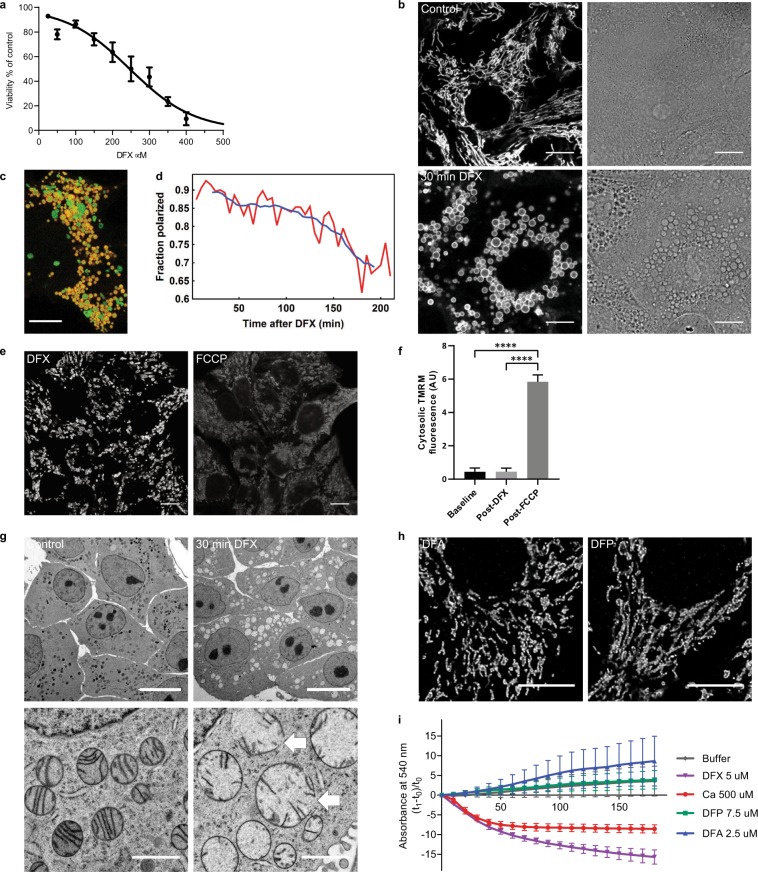
Deferasirox causes acute mitochondrial swelling without depolarization. (**a**) Cell viability in OK cells decreased after 24 hours DFX treatment in a concentration dependent manner (IC_50_ = 246 µM). Line shows log (inhibitor) versus normalized response variable slope analysis (n = 3). (**b**) Live confocal imaging in cells loaded with the ΔΨ_m_-dependent dye TMRM showed that DFX (200 µM) causes acute mitochondrial swelling; after 30 minutes mitochondria remained energized (left), but acquired a distinct rounded shape, which was associated with a change in optical density clearly visible in corresponding phase contrast images (right, scale = 10 µm). (**c,d**) Overlay images of CellLight Mitochondria-GFP BacMam 2.0 (green) and TMRM (red) were used to track the energization state of mitochondria post DFX treatment (scale = 10 µm). The fraction of polarized mitochondria (red line) decreased slowly over time, but even after 3 hours the majority remained energized. Approximately 3,500 mitochondria were sampled at each time point (blue line = the moving average of 40 minutes). (**e,f**) Swollen mitochondria post DFX treatment (20 minutes) showed an abrupt depolarization in response to FCCP (5 µM, 1 minute), with redistribution of TMRM into the cytosol (n = 4, mean ± standard error of the mean [SEM], ****p < 0.0001, one-way ANOVA with Tukey’s multiple comparisons test, scale = 10 µm). (**g**) Electron microscopy images showing mitochondrial swelling, decreased matrix density and rupture of mitochondrial membranes in DFX treated cells (arrows, scale = 10 µm in upper images, 1 µm in lower images). (**h**) Confocal imaging of fixed cells stained with TOM-20 showing no evidence of mitochondrial swelling after 5 hours treatment with the iron chelators deferoxamine (DFA, 200 µM) and deferiprone (DFP, 200 µM, scale = 10 µm). (**i**) Swelling in isolated mitochondria, measured by a decrease in absorption at 540 nm, occurred after 500 µM Calcium or 5 µM DFX, but not after the addition of the iron chelators deferoxamine (DFA, 2.5 µM) or deferiprone (DFP, 7.5 µM). Concentrations for each drug were chosen to take into account relative iron binding ratios. Values were normalized to the first measurement after drug addition (n = 4, mean ± SEM).

We first performed experiments in a well-established PT-derived (OK) cell line^32^. In initial toxicity screens, we ascertained that DFX at a concentration of 200 µM resulted in an approximately 40% decrease in cell viability after 24 hours (Fig. 1a). This concentration was therefore used in further experiments as an appropriate dose to study the mechanism of toxicity (patient blood concentrations are typically in the range 10–100 µM^33^). Using live cell confocal imaging, we observed that DFX induced very rapid and severe swelling of mitochondria (typically within 10 minutes), which underwent a dramatic change in morphology from their characteristic elongated shape to rounded spheres. Surprisingly, during this process mitochondrial membrane potential (Δψ_m_) - visualized using tetramethylrhodamine methyl ester (TMRM) - was maintained (Fig. 1b). However, the optical density in mitochondria was markedly changed in transmission images, signifying water accumulation in the matrix (Fig. 1b). In control cells, elongated mitochondria were observed to be highly mobile, moving extensively along the microtubule network and demonstrating frequent fusion/fission events. In contrast, swollen mitochondria post-DFX were relatively static, displaying an impairment in normal dynamics (Supplementary movie 1).

Co-imaging of TMRM signal with a mitochondrially expressed GFP over longer periods of time revealed that swollen mitochondria eventually lost potential, but even after 3 hours the majority still remained energized (Fig. 1c,d). The maintenance of Δψ_m_ post DFX was supported by immediate dissipation in response to the uncoupler Carbonyl cyanide-4-(trifluoromethoxy)phenylhydrazone (FCCP) (Fig. 1e,f). High-resolution EM confirmed the presence of severe mitochondrial swelling and decreased matrix density following DFX treatment (Fig. 1g). Membrane ruptures were observed in some grossly swollen mitochondria (Fig. 1g), presumably due to hydrostatic pressure, and probably explaining why they can eventually depolarize.

In contrast to DFX, two other iron chelators – deferoxamine and deferiprone – did not have any effect on mitochondrial morphology, even after prolonged incubation (>5 hours), at higher concentrations (up to 1 mM), and in the presence of pluronic to increase cellular uptake (Fig. 1h). Moreover, only DFX induced swelling when the drugs were directly applied to isolated mitochondria (Fig. 1i).

### Deferasirox causes partial uncoupling of the mitochondrial respiratory chain

**Figure 2:**
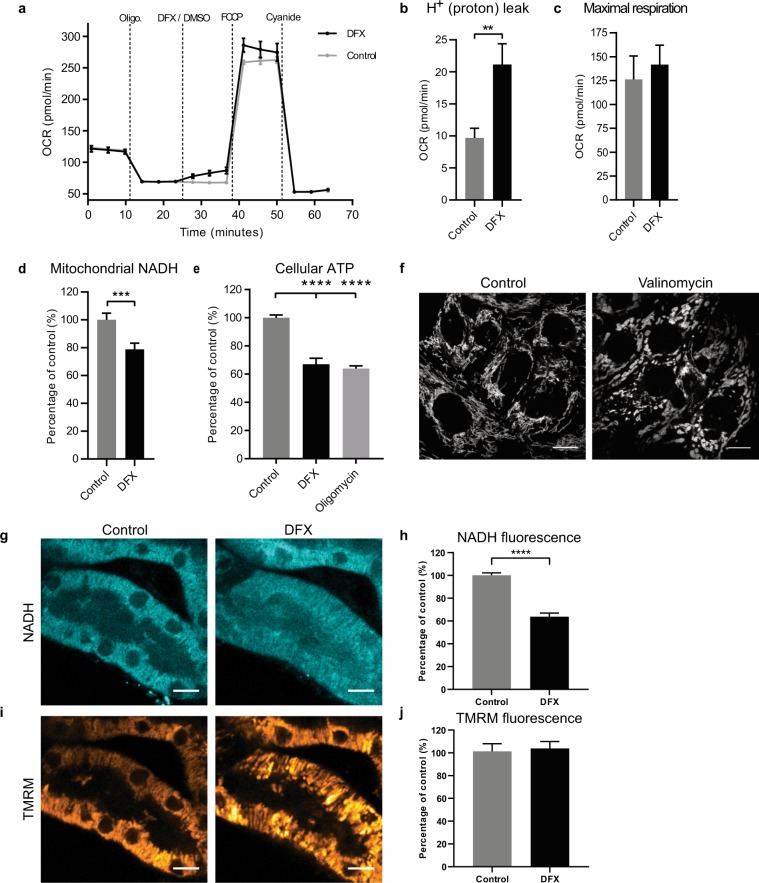
Deferasirox causes partial mitochondrial uncoupling. (**a**) Example raw traces (n = 3 wells per condition, mean ± SEM) of changes in oxygen consumption rate (OCR) in OK cells after addition of DFX (200 µM) or vehicle (DMSO). ATP-linked respiratory chain (RC) activity was inhibited with oligomycin (1 μM). The RC was stimulated with the uncoupler FCCP (5 μM), and inhibited with cyanide (2 mM). (**b**) Cells exposed to DFX (200 μM) had a significantly greater H^+^ (proton) leak than control cells (n = 3 separate experiments, mean ± SEM, unpaired two-tailed *t*-test, **p < 0.01). H^+^ leak = OCR post oligomycin (and DFX/DMSO) – OCR post cyanide. (**c**) No difference was observed in maximal OCR post FCCP (n = 3, mean ± SEM, unpaired two-tailed *t*-test, p = 0.63). (**d**) Live confocal imaging revealed a significant decrease in mitochondrial NADH signal in OK cells 10 minutes after DFX (200 µM) (n = 4, mean ± SEM, unpaired two-tailed *t*-test, ***p < 0.001). (**e**) ATP levels were significantly lower after 30 minutes of treatment with DFX (200 µM), and were similar to cells treated with the ATP synthase inhibitor oligomycin (10 µM) (n = 3, mean ± SEM, one-way ANOVA with Tukey’s multiple comparisons test, ****p < 0.0001). (**f**) Treatment of OK cells with 10 nM valinomycin also produced mitochondrial swelling without depolarization. Representative images are depicted of TMRM loaded cells 30 minutes post valinomycin (n = 3, scale = 10 µM). (**g**,**h**) A decrease in mitochondrial NADH in response to DFX (200 µM) was also observed in proximal tubules in *ex vivo* mouse kidney cortex tissue imaged with multiphoton microscopy (n = 3, mean ± SEM, unpaired two-tailed *t*-test, ****p < 0.0001, scale = 10 µm). (**i,j**) TMRM staining revealed evidence of mitochondrial swelling, but without dissipation of membrane potential (Δψ_m_) (n = 3, mean ± SEM, unpaired two-tailed *t*-test, p = 0.7801, scale = 10 µm).

To investigate functional changes in mitochondria during the acute swelling process induced by DFX, we performed measurements of oxygen consumption rate (OCR) in OK cells. These revealed that after blocking the ATP synthase with oligomycin OCR increased acutely in response to DFX, but maximal OCR achieved post addition of FCCP was similar to control cells, as was the decrease in OCR after adding the RC inhibitor cyanide (Fig. 2a–c). These experiments suggest that DFX increases the proton leak across the IMM (Fig. 2b).

Meanwhile, live confocal imaging revealed that DFX acutely decreased mitochondrial NADH intensity (Fig. 2d), consistent with increased RC activity. Conversely, cellular ATP was significantly decreased following treatment with DFX, to a similar level as in cells exposed to the mitochondrial ATP synthase inhibitor oligomycin (Fig. 2e). Taken together, these findings suggest that DFX does not inhibit the RC, but has an uncoupling effect (i.e. increased RC activity, but decreased ATP). However, this is only partial, and thus not sufficient to depolarize the IMM. This notion was further supported by the finding that a low dose treatment (10 nM) of the known partial uncoupler valinomycin (a mitochondrial potassium ionophore) also produced mitochondrial swelling without depolarization (Fig. 2f).

### Deferasirox induces mitochondrial damage in kidney cortex tissue

PT-derived cell lines are recognized to have limitations as experimental models^34^. We therefore sought to know whether DFX also induces a similar phenotype in native PTs. To address this, we performed live imaging of freshly cut sections of mouse kidney cortex, using multiphoton microscopy and protocols that we established in previous studies^35^. DFX was applied at 200 µM directly to the tissue, under tightly controlled experimental conditions. As in the OK cells, in response to DFX we observed rapid swelling of mitochondria in PTs. Mitochondria remained energized during the swelling process, but displayed a decrease in NADH fluorescence signal, consistent with RC uncoupling (Fig. 2g–j).

### Deferasirox induces mitochondrial swelling in the kidney proximal tubule *in vivo*

**Figure 3:**
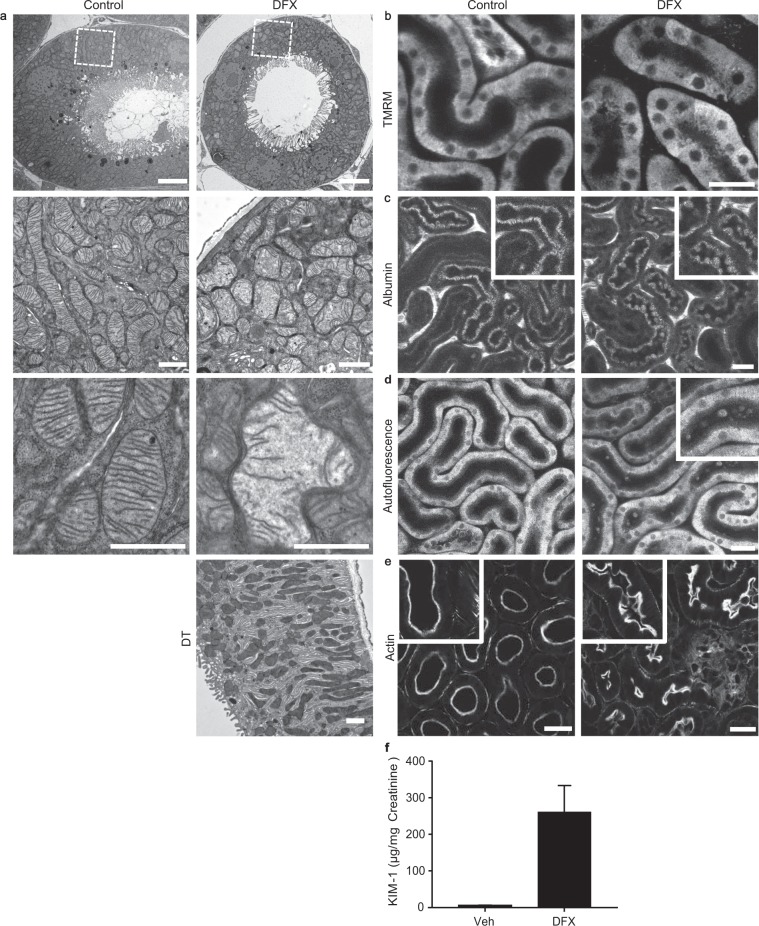
Deferasirox induces mitochondrial swelling and cellular damage in the kidney proximal tubule *in vivo*. (**a**) Mice were treated with DFX (100 mg/kg) or vehicle once daily for a total of 10 days. Electron microscopy images confirmed the existence of swollen mitochondria with decreased matrix density in PTs of DFX treated mice (n = 2), but not in distal tubules (DT), or in vehicle controls (n = 2; scale = 5 µm in upper panel, scale = 1 µm in other images). (**b**) Intravital multiphoton microscopy revealed that mitochondrial TMRM signal was markedly heterogeneous in the PTs of DFX treated animals, consistent with variation in ΔΨ_m_. Moreover, in contrast to vehicle treated animals, the normal elongated appearance of individual mitochondria could no longer be discerned (scale = 30 µm). (**c**) Fluorescently labeled albumin was injected intravenously, and subsequently filtered and taken up by endocytosis to label PTs. Damaged PT cells in DFX treated animals were observed to protrude into the tubular lumen (scale 30 µm). (**d**) Cellular debris was visible in PT lumens in the blue auto-fluorescence channel in DFX-treated mice, consistent with shedding from damaged cells (scale = 30 µm). Data shown in B-F are representative for DFX-treated (n = 10) and vehicle-treated mice (n = 9). (**e**) Tissue cryo-sections from kidney cortex were stained for the actin-enriched apical brush border and also revealed evidence of cellular protrusion in PTs of DFX-treated mice (scale = 20 µm). (**f**) Spot urine KIM-1/creatinine ratio was significantly increased in DFX exposed animals (n = 5) compared to vehicle controls (n = 6). Data depicted are mean values ± SEM (Mann-Whitney non-parametric test, **p < 0.01).

To investigate the effects of DFX on mitochondria in the kidney *in vivo*, mice were given DFX (100 mg/kg bodyweight) once daily as an intraperitoneal injection, for a total of ten days. EM images showed clear evidence of mitochondrial swelling and decreased matrix density in PTs, but not distal tubules, in DFX-exposed mice (Fig. 3a). To assess effects on mitochondrial function, we performed intravital multiphoton microscopy in anaesthetized mice using protocols established previously^36^. We observed abnormal heterogeneity in Δψ_m_ in PTs of treated mice, and individual mitochondria could no longer be resolved due to swelling (Fig. 3b). Damaged PT cells protruded apically into the tubular lumens which contained visible cellular debris (Fig. 3c–e). Meanwhile, urinary levels of KIM-1, a widely used biomarker specific for PT toxicity, were much higher in DFX treated animal than controls (Fig. 3f). Thus, the mitochondrial phenotype we observed with DFX *in vitro* also occurs in PTs *in vivo*, and is associated with significant cellular damage.

### Deferasirox-induced mitochondrial swelling is independent of permeability transition pore opening

**Figure 4:**
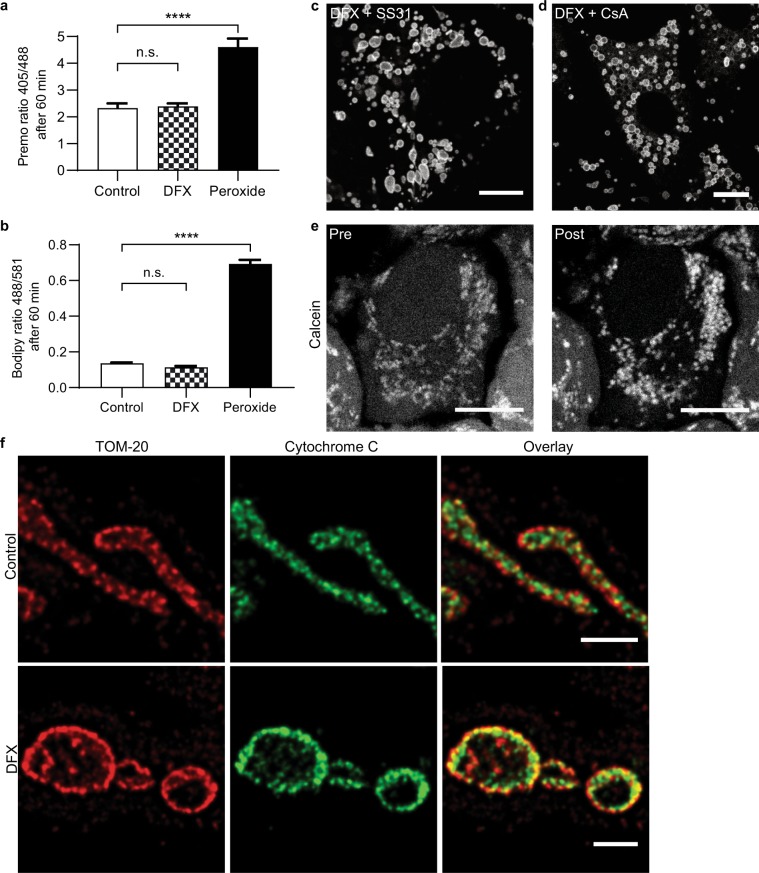
Deferasirox-induced swelling is independent of the mitochondrial permeability transition pore. (**a**) Cellular levels of ROS were measured using the ratiometric sensor Orp1-roGFP after 1 hour of treatment, and were not elevated in DFX (200 µM) treated OK cells. H_2_O_2_ was added directly to cells as a positive control (n = 4 experiments, total of 32 cells, mean ± SEM, one-way ANOVA with Tukey’s multiple comparisons test, ****p < 0.0001). (**b**) Lipid peroxidation after 1 hour treatment was measured using Bodipy 581/591 C11, and was also not increased in DFX (200 µM) treated cells (n = 4 experiments, total of 16 cells, mean ± SEM, one-way ANOVA with Tukey’s multiple comparisons test, ****p < 0.0001). (**c,d**) Neither the antioxidant SS-31 (500 µM) nor the mPTP inhibitor cyclosporin A (CsA, 1 µM) prevented subsequent DFX-induced mitochondrial swelling (scale = 10 µm). (**e**) Calcein signal remained localized to mitochondria after 25 minutes of treatment with DFX (200 µM, scale = 10 µm). (**f**) Cos-7 cells treated with 10 minutes of DFX (200 µM) were stained for TOM-20 and cytochrome c, and imaged with STED super-resolution microscopy. Mitochondria in DFX treated cells were swollen, but cytochrome c remained within the organelles (scale = 1 µm).

Next, we performed experiments to investigate the underlying mechanism of DFX-induced mitochondrial swelling in cells. Whilst a small degree of mitochondrial swelling can occur in response to a number of insults^37^, severe swelling in pathological states is classically attributed to the opening of a large non-specific pore (the mPTP) in the IMM, typically in the presence of oxidative stress, leading to immediate dissipation of Δψ_m_ and release of cytochrome c into the cytosol^9^. However, given that mitochondria remained energized during the swelling process, we suspected that the effects of DFX were not due to opening of the mPTP. In support of this notion, we found no evidence that DFX increases ROS production or lipid peroxidation in OK cells (Fig. 4a,b), and DFX-induced swelling was not inhibited by the mitochondrial targeted anti-oxidant SS-31^38^ (Fig. 4c). Moreover, mitochondrial swelling was also not prevented by cyclosporin, a known inhibitor of the mPTP (Fig. 4d). In addition, we did not detect mitochondrial release of the small fluorescent protein Calcein (Fig. 4e), which is widely used to assess mPTP opening in living cells^39^. To assess cytochrome c distribution post-DFX, we performed experiments in kidney derived Cos7 cells, as we were unable to achieve successful antibody staining in OK cells, probably due to species differences. We confirmed that DFX also induced rapid swelling of mitochondria in Cos7 cells, but this was not associated with any detectable release of mitochondrial cytochrome c (Fig. 4f). Thus, taken together, our findings suggest that DFX-induced swelling is independent of mPTP opening.

### Deferasirox-induced mitochondrial swelling occurs due to an influx of water

**Figure 5:**
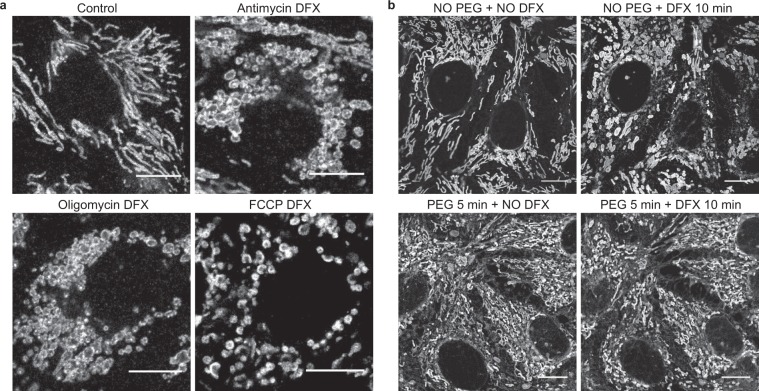
Deferasirox-induced mitochondrial swelling is driven by osmotic gradients independent of respiratory chain activity. (**a**) TOM-20 staining of mitochondria in OK cells pre-treated for 10 minutes with the respiratory chain complex III inhibitor antimycin A (2.5 µM), or the ATP synthase inhibitor oligomycin (10 µg/ml), or for 1 minute with the uncoupler FCCP (10 µM). None of these interventions prevented mitochondrial swelling after the subsequent addition of DFX (200 µM, scale = 10 µm). (**b**) Live confocal imaging of OK cells loaded with MitoTracker Deep Red FM. Treatment with DFX (200 μM) alone caused mitochondrial swelling, as expected. In contrast, mitochondrial swelling was prevented in cells incubated with 10% PEG 400 for 5 minutes prior to DFX exposure, to increase cytosolic osmolarity (scale = 10 μM).

The normal activity of the RC generates ionic gradients across the IMM and produces water within the mitochondrial matrix^40^. However, we found that DFX-induced mitochondrial swelling was not prevented by agents that either inhibit or uncouple the RC (Fig. 5a). In contrast, when we acutely increased the cytosolic osmolarity in cells by incubating cells for 5–10 minutes in 10% polyethylene glycol (PEG) - an inert substance that rapidly draws water out of cells by osmosis^41^ - we found that mitochondrial swelling was prevented (Fig. 5b). These results suggest that DFX-induced mitochondrial swelling occurs due to an influx of water into the matrix, but that this is driven by ionic movements and/or osmotic gradients across the IMM that are independent of RC activity or Δψ_m_.

### Deferasirox-induced mitochondrial swelling is reduced by binding to iron or albumin

**Figure 6:**
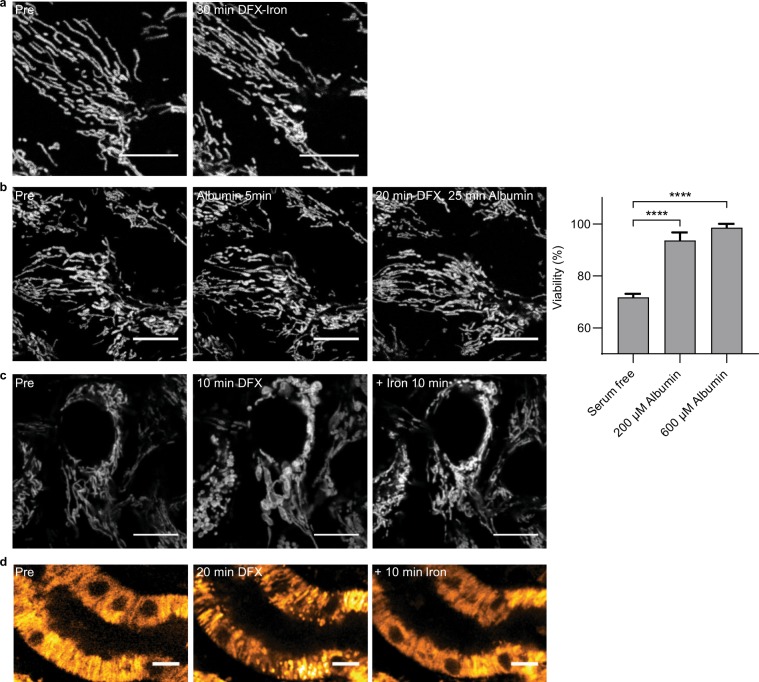
Deferasirox-induced mitochondrial swelling is caused by the free drug and is reversible. (**a**) In OK cells loaded with TMRM, addition of Iron (III) (200 µM) simultaneously with DFX (200 µM) prevented acute mitochondrial swelling (scale = 10 µm). (**b**) Pre-treatment for 5 minutes with albumin (200 µM) also prevented mitochondrial swelling, and preserved cell viability after 24 hours treatment with DFX (n = 3, mean ± SEM, one-way ANOVA with Tukey’s multiple comparisons test, ****p < 0.0001, scale = 10 µm). (**c**) Addition of Iron (III) (200 µM) 10 minutes post DFX (200 µM) caused reversal of mitochondrial swelling in TMRM loaded cells (scale = 10 µm). (**d**) Reversibility of mitochondrial swelling with Iron (III) (200 µM) was also observed in kidney cortex slices loaded with TMRM (scale = 10 µm).

To investigate whether it is the free or chelated form of the drug that causes damage, we incubated cells with DFX and iron, and found that this prevented toxicity (Fig. 6a). Moreover, DFX is over 99% protein bound *in vivo*^26^, and we observed that incubation with albumin also prevented acute mitochondrial swelling, and improved cell viability at 24 hours (Fig. 6b). These results suggest that it is the free drug that is responsible for the toxic effect, and that protein binding might modulate toxicity *in vivo*.

### Swollen mitochondria can recover post deferasirox treatment

Next, we explored whether mitochondria swollen by DFX can reform their normal shape. Since DFX has a very strong affinity for iron we added it to the medium to complex DFX and therefore render it non-toxic. Following the addition of iron, we found that mitochondria gradually reformed their normal elongated morphology (Fig. 6c, Supplementary movie 2), showing that DFX-induced mitochondrial swelling is reversible, and suggesting that mitochondria have a mechanism to extrude water from the matrix. The same reversibility was also observed in tissue slices (Fig. 6d).

### Deferasirox directly alters membrane integrity in an artificial model of the inner mitochondrial membrane

Since we found no evidence that DFX induces mPTP opening and that it is the free drug that causes toxicity, we considered that it might exert direct effects on the integrity of the IMM, leading to a change in permeability. Of note, DFX is highly lipophilic (partition coefficient [LogP] 3.52–4.74, Drugbank), so could insert into lipid membranes. To explore this hypothesis, and exclude the possibility that DFX might instead interact with certain mitochondrial proteins, we performed experiments in artificial giant lipid vesicles. These were prepared to contain phospholipids (POPC and POPE), cardiolipin, and cholesterol (in a 40:30:25:5 ratio), to accurately model the consistency of the IMM.

**Figure 7:**
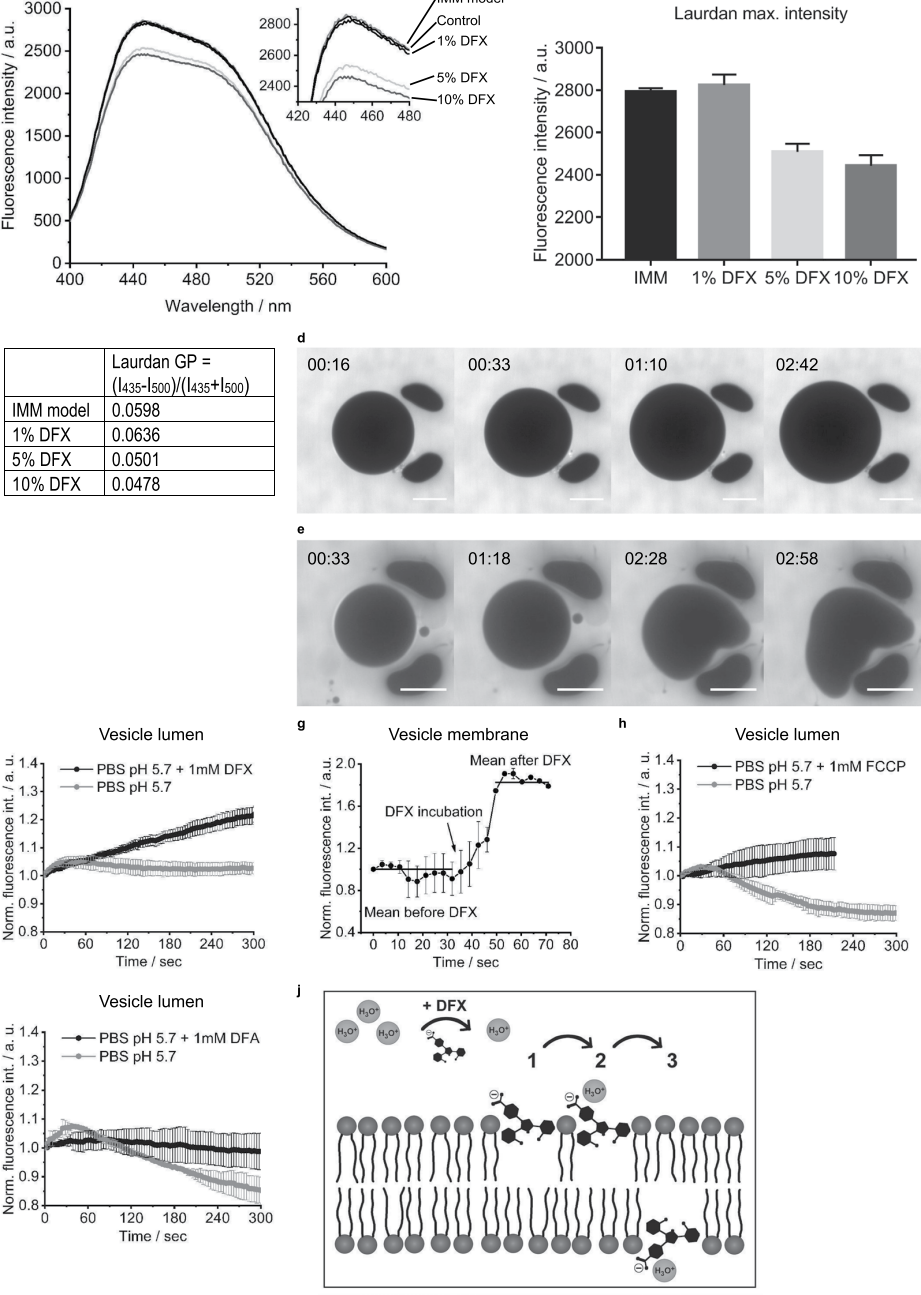
Deferasirox exerts direct effects on membrane integrity in a lipid vesicle model of the inner mitochondrial membrane. (**a**) Spectra of IMM liposomes; POPC/POPE/Cardiolipin/Cholesterol (40:30:25:5) prepared in presence of Laurdan (500:1), DFX and DMSO (control). Mean of n = 3. (**b**) Fluorescence maximal intensity deduced from 3 measurements. (**c**) Laurdan generalized polarization (GP). The slightly reduced values at high DFX concentrations indicate a minor reduction of membrane order. (**d,e**) Giant IMM model vesicles, prepared in PBS, captured in a trap-chamber in presence of 100 µM AF488-Carboxylic acid and PBS:ddH_2_O (1:1, v-v) (AF488 fluorescence image in grayscale). Mild osmotic swelling was observed in control vesicles, but normal morphology was maintained (**d**). In contrast, incubation with DFX lead to loss of membrane stability, but without leakage of dye (**e**). (**f**) Giant IMM vesicles were prepared in PBS pH 7.4 containing 0.8 mg/ml pHrodo, captured in a trap-chamber and incubated with PBS pH 5.7 containing 0.8 mg/ml pHrodo, followed by PBS pH 5.7 containing 1 mM DFX and 0.8 mg/ml pHrodo. Measurement of the vesicle lumen signal (n = 3). (**g**) Measurement of the intensity of membrane-bound pHrodo. The timeframe covers the moment when DFX reaches the vesicle membrane (arrow) (n = 2). (**h,i**) Measurement of the vesicle lumen pHrodo signal with 1 mM FCCP (n = 3) and 1 mM deferoxamine (DFA) (n = 2). (**j**) Proposed mechanism of action of DFX on the IMM. DFX contains polar groups and is negatively charged at physiological pH, which likely enhances water, protons and H_3_O^+^ ions at the outer surface. DFX possibly interacts with polar groups in the phospholipid head group region of the bilayer and the phenol moieties of DFX support partial insertion into the hydrophobic membrane region (1). This enables DFX to bring water, protons and H_3_O^+^ ions into close contact with lipid head groups (2), increasing the probability of translocation (3), but without changing permeability to other molecules. In addition, as a drug with a membranophilic logP it might also undergo a flip-flop across the bilayer leaflets and inherit a protonophore character.

First, we investigated the effect of DFX on membrane order and fluidity using the dye Laurdan^42^. We observed a small decrease in Laurdan intensity at higher DFX concentrations, but no evidence of a major spectral shift (Fig. 7a,b). Instead, a small decrease in Laurdan generalized polarization (GP), a measure for the phospholipid order^43^, was observed for high DFX concentrations (Fig. 7c). These findings suggest that DFX only intercalates weakly into the hydrophobic part of the membrane, but that it brings water, protons and hydronium (H_3_O^+^) ions into close proximity, leading to partial quenching of the probe signal^42^.

Next, to investigate the effect of DFX on membrane permeability, vesicles were prepared in PBS and captured in a trap chamber to perform real-time imaging studies. They were then incubated with DFX and the fluorescent dye AF488-Carboxylic acid (molecular weight 885 g/mol) in a hypo-osmolar solution (PBS:ddH_2_O; 1:1). We observed that vesicles swelled under these conditions and became distorted, consistent with a change in membrane integrity (Fig. 7d,e). However, no dye was observed to enter the vesicles, showing that permeability to small molecules is not increased by DFX (Fig. 7d,e). To investigate the effect of DFX on membrane permeability to protons, vesicles were prepared in PBS containing the pH sensitive dye pHrodo. Upon decreasing the extra-vesicular pH to 5.7, to mimic the normal proton gradient across the IMM, only a small decrease in intra-vesicular pH was noted when DFX was absent (Fig. 7f), suggesting relative impermeability to protons. However, in the presence of DFX a much bigger decrease in intra-vesicular pH was observed (Fig. 7f–h). The most striking change occurred in the immediate vicinity of the membrane (Fig. 7g, Supplementary movie 3), closely matching the TMRM signal pattern observed previously in swollen mitochondria within DFX treated cells. In contrast, deferoxamine did not produce the same effect in lipid vesicles (Fig. 7i).

In summary, these data show that DFX exerts a direct, but subtle change in the integrity of the IMM, leading to an increase in permeability to protons, but not other molecules. This most likely explains why DFX causes changes in mitochondrial morphology and partial uncoupling in cells, but without depolarization.

## Discussion

DFX is a frequent cause of organ toxicity in patients for reasons that were previously unclear. We have found that DFX causes severe mitochondrial swelling and partial uncoupling of the RC, thus explaining why this drug is more harmful than other clinically used chelators. Importantly, DFX-induced mitochondrial swelling occurs in the absence of depolarization or mPTP opening. These findings have potentially important implications for understanding mechanisms of drug toxicity in humans and the pathogenesis of mitochondrial swelling in disease states.

The mechanisms via which mitochondrial volume is regulated within cells remain poorly understood. For example, there is controversy concerning whether mitochondria express aquaporin water channels in the IMM^44–46^. Nevertheless, due to a high surface to volume ratio, isolated mitochondria typically display rapid osmotic equilibration^46^. Loss of volume homeostasis and mitochondrial swelling is a common occurrence in pathological states, and is often attributed to mPTP opening. However, we reasoned that DFX-induce mitochondrial swelling is likely to be independent of mPTP opening, since Δψ_m_ dissipation is the *sine qua non* of this phenomenon^47^. Instead, using an *in vitro* lipid vesicle system we have shown that DFX induces a direct, but subtle effect on the integrity of the IMM, leading to increased permeability of protons, but not other molecules. This experimental approach has several potential advantages over the traditional strategy of using isolated mitochondria. For example, it eliminates confounding effects of membrane proteins, and avoids the damage that can occur to the IMM during the isolation process^48,49^, which might obscure detection of more nuanced effects. Moreover, vesicle morphology and size can be assessed directly, rather than relying on indirect readouts like light scattering^50^. However, it is important to note that the isolated mitochondria model also offers some distinct benefits, such as the ability to assess bioenergetics and the activity of specific transporters^51^.

As a highly lipophilic drug, DFX most likely partially inserts into the hydrophobic part of the IMM, but without causing sufficient membrane disorder to induce a large generalized increase in permeability to small molecules. Since it contains polar groups (OH, COOH) and is negatively charged at physiological pH it may favor enrichment of water, protons and H_3_O^+^ in the vicinity, thus specifically enhancing the movement of protons and water across the IMM (Fig. 7j). The movement of the former would explain the partial RC uncoupling effect that we observed with DFX in cells. However, established RC uncouplers like FCCP do not typically cause severe mitochondrial swelling^4^. Furthermore, pre-treatment of cells with RC inhibitors or FCCP to dissipate Δψ_m_ did not prevent DFX-induced swelling. Thus, it is likely to be the movement of water rather than protons that predominantly explains this phenomenon, a notion supported by the fact that increasing cytosolic osmolarity with PEG prevented DFX-induced mitochondrial swelling.

At present, we can only speculate what drives the movement of water into the mitochondrial matrix. Previous studies have suggested that intracellular K^+^ gradients might play a key role in mitochondrial volume regulation^4,51^, and increases in IMM K^+^ permeability induce mitochondrial swelling^4^. In response to an influx of K^+^ the mitochondrial K^+^/H^+^ exchanger is activated and can maintain Δψ_m_, at the cost of partial uncoupling of the RC^4^. Thus, K^+^ influx could underlie DFX-induced mitochondrial swelling, perhaps due to the activation of mitochondrial K^+^ channels^52^. However, although we could partially recreate the DFX phenotype with the potassium ionophore valinomycin, we could not produce such severe mitochondrial swelling without inducing IMM depolarization. Moreover, the K^+^/H^+^ exchanger requires the existence of normal proton gradients across the IMM to work, and valinomycin-induced mitochondrial swelling is prevented by inhibiting or uncoupling the RC^53^. In contrast, prior treatment with RC inhibitors/uncouplers did not prevent DFX-induced mitochondrial swelling. Furthermore, DFX directly induced structural and functional changes in lipid vesicles in the absence of any K^+^ channels or gradients. Thus, while K^+^ could play a role, there may be additional forces that drive water movement across the IMM. For example, since mitochondria actively import many nuclear encoded proteins^54^, this might produce a high inward-directed oncotic pressure, but further experiments will be required to confirm or refute this.

Our findings raise the possibility that other drugs with chemical similarities to DFX might also exert effects on the IMM. Of note, DFX is synthesized from salicylic acid, which is a non-steroidal anti-inflammatory drug (NSAID). NSAIDs also cause clinically significant organ toxicity in humans and induce mitochondrial swelling, which has previously been attributed to mPTP opening^55,56^, but our results provide an alternative explanation. Moreover, in high-throughput screens a number of different lipophilic weak acids have been identified that can cause partial RC uncoupling^57^, but to what extent they also affect mitochondrial volume remains unclear.

This study also has some important clinical implications for understanding DFX toxicity in patients. Firstly, since DFX-induced mitochondrial swelling is an off-target effect unrelated to iron metabolism this explains why other chelators do not produce similar toxicity. Secondly, in mice chronically exposed to DFX, we observed mitochondrial swelling and cellular damage in kidney PTs, suggesting that the toxic mechanisms identified *in vitro* also occur *in vivo*. The reasons why DFX nephrotoxicity is exclusively limited to the PT are currently unclear, but are likely related to the existence of specific uptake mechanisms^58^. Thirdly, a decrease in the efficiency of ATP production due to partial mitochondrial uncoupling could cause functional defects in PT solute transport that are typically observed in patients with DFX toxicity^26^. Finally, risk factors for DFX toxicity in humans are not yet well established. We observed that binding of DFX to iron or albumin reduced toxicity *in vitro*, suggesting that patient iron and nutritional status might play roles in determining baseline risk of toxicity.

There are several potential limitations to our study. First, experiments were performed at a slightly higher DFX concentration than is typically found in patients^33^. However, since PT cells *in vivo* accumulate drugs efficiently from the blood via organic transporters^59^, higher doses may be required with *in vitro* PT models to more accurately reflect the situation in living animals. Second, it is possible that DFX induces small changes in Δψ_m_ that we could not detect using TMRM. Third, although we observed evidence of mitochondrial swelling *in vivo*, this was not as severe as *in vitro*, so we cannot be sure that the mechanisms are the same. Individual mitochondria in the PT are closely surrounded by extensive infoldings of the basolateral cell membrane^60^, which could limit the scope for volume expansion. It is also conceivable that in a chronic model mitochondria might partially adapt to an influx of water. Fourth, we cannot exclude the possibility that DFX decreases cellular ATP via an effect on glycolysis, although PT cells *in vivo* are almost entirely dependent on aerobic metabolism^27^. Finally, the exact factors that drive DFX-induced water influx remain to be elucidated.

In summary, by elucidating the previously unknown cellular mechanism of toxicity from the iron chelator DFX we have found evidence that subtle alterations in IMM properties can produce major changes in mitochondrial volume within cells without mPTP opening or depolarization. These findings underline that therapeutic drugs can have biophysical side effects unrelated to their intended mechanism of action, and that understanding the nature of these can yield important new insights into cellular physiology.

## Methods

### Animal studies

All experiments were performed on 8 to 12 week old male C57BL/6JRj mice. For the *in vivo* study, animals received a single daily intra-peritoneal injection of 100 mg/kg bodyweight DFX (Biochempartner), dissolved in saline and 0.16% Tween 20 (Thermo Fisher Scientific), for a total of ten days. Vehicle controls were injected with saline and Tween alone. All animal experiments were carried out in accordance with the guidelines and regulations of the Zurich Cantonal Veterinary Office. All experimental protocols were approved by the Zurich Cantonal Veterinary Office (Ref: ZH021/15).

### Cell lines

Opossum kidney (OK) cells were a kind gift from the group of Prof O. Devuyst (Physiology, University of Zurich). Cos-7 cells were a kind gift from the Center for Microscopy and Image analysis at The University of Zurich. To label mitochondria with GFP, cells were incubated for 2 hours with CellLight Mitochondria-GFP BacMam 2.0 (5 particles per cell) and BacMam enhancer (both Thermo Fisher Scientific) one day prior to experimentation. For the detection of intracellular H_2_O_2_, cells were exposed to Premo Cellular Hydrogen Peroxide Sensor Orp1-roGFP and BacMam enhancer 24 hours before experiments.

### Immunofluorescence in cells

Cells were fixed with 3% paraformaldeyde and permeabilized with 0.1% TritonX-100 (Surfact-Amps detergent solution; Thermo Fisher Scientific), and blocked with 1% BSA. For confocal imaging they were additionally blocked with donkey serum before incubation with primary antibody (1:150, TOM-20, rabbit polyclonal, Santa Cruz, sc11415) overnight at 4 °C, and the secondary antibody (1: 500, Alexa 488, donkey anti rabbit, Jackson Immuno Research) was incubated for 2 hours before mounting with DAKO glycergel mounting medium. For Stimulated Emission Depletion (STED) super-resolution microscopy cells were incubated with the primary antibodies (1:125 TOM-20, 1:50 Cytochrome c, mouse monoclonal clone 6H2.B4, Biolegend) overnight at 4 °C, and the secondary antibodies (1:200 Atto 594, goat anti mouse, Sigma; 1: 200 Abberior Star 635, goat anti rabbit, Abberior GmbH) were incubated for 2 hours before mounting with ProLong Diamond antifade mountant (Thermo Fisher Scientific). Confocal and STED microscopy were performed on a Leica SP8 inverse STED 3×. For gated STED imaging the dyes were excited at 585 nm and 635 nm, respectively. The emission was collected on HyD detectors with filter settings 590–628 nm and 659–751 nm, using time gates of 1–6 ns and 0.8–6 ns, respectively. For both dyes the 775 nm STED depletion laser was used at 100%.

### Staining in fixed kidney tissue

Kidney tissue was fixed via the aorta with 3% paraformaldehyde in a phosphate buffered solution. Brush-border actin filaments were stained in 5 µm thick cryosections with ActinRed 555 ReadyProbes reagent (Invitrogen, R37112) according to manufacturer’s instructions. Images were acquired using a Leica SP8 upright confocal microscope.

### Live imaging

Cells were seeded on Poly-L-lysine coated coverglasses (Hecht-Assistent) and grown to 90% confluence. Imaging was performed at 37 °C in a buffer adjusted to pH 7.4 containing (in mM): 138 NaCl, 5.6 KCl, 1.2 NaH_2_PO_4_, 2.6 CaCl_2_, 1.2 MgCl_2_, 10 Glucose, 4.2 NaHCO_3_ and 10 HEPES. All dyes were purchased from Thermo Scientific, with the exception of SiR tubulin (Spirochrome), and were used in the following concentrations: TMRM 50 nM (excitation 555 nm), Mitotracker Deep Red FM 200 nM (excitation 644 nm), SiR tubulin 2 µM (excitation 650 nm, used with verapamil 10 µM for enhancement of loading), Calcein-AM 250 nM (excitation 488 nm, used with 2.5 mM probenecid) and Bodipy 581/591 C11 2 µM (excitation 510 nm and 580 nm). Confocal microscopy was performed on a Leica SP8 inverse STED 3×. For the detection of NADH a Leica SP5 confocal microscope equipped with a 355 nm UV was used. For analysis of mitochondrial signals regions of interest were drawn around mitochondrial dense parts of cells. Redistribution of TMRM post mitochondrial depolarization was detected by drawing regions of interest in the cytosolic compartment.

Kidney cortex tissue slices were generated from freshly externalized organs in mice anaesthetized with intra-peritoneal ketamine (0.065 g/kg) and xylazine (0.01 g/kg), according to previously established protocols^35^. After the removal of the capsule one pole of the kidney was mounted and cut with a vibratome (Microm HM 650 V, Thermo Scientific) into 250 µm thick sections. The tissue was kept until usage at 4 °C in a physiological buffer adjusted to pH 7.4 and gassed with carbogen (95% O_2_/5% CO_2_), containing (in mM): 118 NaCl, 4.7 KCl, 1.2 KH_2_PO_4_, 1.8 CaCl_2_, 1.44 MgSO_4_, 5 glucose, 10 NaHCO_3_, 10 HEPES, 5 pyruvate, 2.5 sodium butyrate and 2.5 sodium lactate. For live imaging slices were mounted into a heated chamber (Warner Instruments) containing oxygenated buffer. Imaging was performed with an Olympus Fluoview 1000 MPE equipped with a XPlan N 25×/1.05 objective, coupled to an ultrafast Ti:Sapphire laser system. NADH was excited at 720 nm. For the measurement of Δψ_m_ slices were incubated for 1 hour before experiments in TMRM (50 nM), which was excited at 850 nm. To analyze NADH and TMRM signals, regions of interest were drawn around mitochondrial rich regions of cells.

Intravital imaging was performed as described previously^61^. In brief, mice were anesthetized with inhaled isoflurane (Attane, Provet AG) in 100 ml/min oxygen. The internal jugular vein was cannulated for dye application and the left kidney was externalized for imaging. Animals were placed on a custom-built temperature-controlled holder and imaging was performed on a custom-built microscope (described in^62^), equipped with a broadband tunable laser (Insight DS Dual, Spectraphysics). For the visualization of mitochondria 30 µg TMRM was injected in 120 µl 0.9% NaCl, while 600 µg Alexa 488 labeled albumin (Thermo Fisher Scientific) was injected in 120 µl 0.9% NaCl to assess tubular protein handling. An XPlan N 25×/1.05 water immersion objective (Olympus) was used for imaging. Both dyes were excited at 800 nm and the emission light was collected with bandpass filters 525/50 and 605/50.

### Electron microscopy

OK cells were grown on sapphire disks and were high pressure frozen, processed and embedded as described previously^63,64^. For imaging of kidney tissue, anesthetized mice were perfused with 3% paraformaldehyde in 0.1 M phosphate buffer, via the abdominal aorta. Kidneys were fixed in 2.5% glutaraldehyde in 0.1 M phosphate buffer at 4 °C, followed by 1% OsO_4_ in cacodylate buffer. After contrast enhancement with 1% uranylacetate and dehydration, samples were embedded in Epon/Araldite. 70 nm thick sections were cut on an Ultramicrotome (Reichert Ultracut) and images were acquired with an FEI CM 100 equipped with a Gatan Orius 1000 camera.

### Cell viability

Cell viability was assessed according to an established protocol^65^. In brief: cells were seeded in a 96 well plate and treated for 24 hours. They were then incubated for 2 hours at 37 °C with Neutral Red medium, washed with PBS and lysed with ethanol (50%), water (49%), glacial acid (1%), before optical density was measured at 540 nm (Synergy 2 Multidetection reader, Biotek). Values were normalized to control after subtraction of the blank.

### Oxygen consumption

Cells were plated in Seahorse XFp 8 well culture plates one day prior to experiments. 1 hour before analysis cells were incubated at 37 °C without CO_2_, in the same buffer used for live imaging experiments. Cartridges were filled with stock solutions of drugs dissolved in the buffer. After each injection oxygen consumption was measured in 3 cycles, each consisting of 2 minutes mixing followed by 2 minutes of measurement.

### ATP determination assay

Cells were grown to 90% confluence and incubated with DMSO (0.2%), DFX (200 μM) or oligomycin (10 μM) for 30 minutes at 37 °C. They were washed once with pre-warmed PBS and lysed for 5 minutes on ice using CelLytic M (C2978, Sigma). Cells were centrifuged to collect the supernatant and intracellular ATP was quantified using an ATP determination kit (A22066, Invitrogen), following the manufacturer’s instructions. Luciferin-luciferase bioluminescence was measured using a multi-mode microplate reader (Synergy 2, Biotek). Protein concentration was determined using the Quick Start Bradford assay (Bio-Rad), with BSA as the standard. Intracellular ATP concentrations were normalized to protein content and expressed as a percentage of control cells.

### Lipid vesicle experiments

Liposomes for fluorescence measurements were prepared using an established protocol^42^. Briefly, lipids, DFX and Laurdan (500:1) were mixed in molar ratio; a lipid film was dried under a stream of nitrogen and vacuum for 2 hours. Liposomes (0.6 mM) were swollen in phosphate buffered saline, pH 7.4 (PBS) at 60 °C under vigorous vortexing and extruded, 31×, 200 nm, (Avestin Liposofast, Ottawa, Canada) at 60 °C. The control contained DMSO without DFX in the same amount as used for the maximal DFX concentration. Laurdan fluorescence was excited at 360 nm and emission spectra were recorded at 22 °C between 380 and 600 nm with a slit of 1 nm.

Giant vesicles for measurements of proton permeability were prepared in PBS pH 7.4 containing 0.8 mg/ml pHrodo by gentle swelling as previously reported^66^, trapped in a microfluidic chamber and incubated with DFX or DFA and pHrodo at 28 °C. Control experiments containing DMSO did not alter the results (data not shown). Fluorescence intensity was analyzed using FIJI/ImageJ at equatorial sections of confocal laser scanning microscopy images (raw data) and contrast enhanced for presentation (images, video). Since pHrodo accumulated on the rim, to record changes in luminal pH regions of interest were drawn in the center of the vesicle.

### Swelling in isolated mitochondria

Mitochondria were isolated from 1 × 10^8^ OK cells using the Qproteome Mitochondria Isolation Kit (Qiagen), according to manufacturer’s instructions. Mitochondrial swelling was measured as described previously^67^. In brief, freshly isolated mitochondria were resuspended in reaction buffer containing 150 mM sucrose, 50 mM potassium chloride, 2 mM KH_2_PO_4_, 5 mM succinic acid, 100 μM NADH, and 5 mM HEPES adjusted to pH 7.4. The concentration of mitochondria was determined using Bradford Protein Assay (Bio-Rad) and adjusted to 0.5 mg per ml. After 3 minutes of equilibrium, buffer, calcium (500 µM) or iron chelators (at equivalent doses according to iron binding ratio) were added. Mitochondrial swelling was monitored by measuring the absorbance at 540 nm every 10 seconds for 3 minutes, using a Libra S70 double beam spectrophotometer (Biochrom).

### Urinary KIM-1 analysis

Spot urine KIM-1 urine levels were determined using an ELISA kit (MKM100, R&D Systems), according to the manufacturer’s instructions, and were normalized to creatinine, which was measured with a UniCel DxC 800 Synchron Clinical System (Beckman Coulter).

### Statistical analysis

Data from confocal and STED imaging was deconvolved using Huygens Professional software (Scientific Volume Imaging B.V.). Image processing for all microscopy data was done in FIJI and Imaris 8.3 (Bitplane AG). Statistical analysis was performed using Graph Pad Prism. To investigate the effect of DFX on mitochondrial energization over time, 36 randomly acquired images containing mitochondrial signals (representing approximately 3,500 individual mitochondria) from multiple cells were analyzed per time point. The GFP-mitochondrial channel of the images was segmented using supervised machine learning (Ilastik: Interactive learning and segmentation toolkit, 10.1109/ISBI.2011.5872394) to identify individual mitochondria and the corresponding TMRM channel intensities were then extracted using FIJI. The obtained fluorescence intensity data was further analyzed and visualized using Mathematica (Wolfram Research, Illinois). The bimodal distribution of the TMRM signal allowed the calculation of a polarization survival probability using fixed thresholds.

## Supplementary information

Supplementary information.

Movie 1.

Movie 2.

Movie 3.
