# Supplementary material for: The iron chelator Deferasirox causes severe mitochondrial swelling without depolarization due to a specific effect on inner membrane permeability
Source: Sci Rep. 2020 Jan 31;10:1577. doi: 10.1038/s41598-020-58386-9 (PMC6994599; doi:10.1038/s41598-020-58386-9)
Supplement: Supplementary file 1 — Supplementary information. [file 41598_2020_58386_MOESM1_ESM.pdf]

Supplementary Materials

The iron chelator Deferasirox causes severe mitochondrial swelling without depolarization due to a specific effect on inner membrane permeability

Esther M Gottwald[1], Claus D Schuh[1], Patrick Drücker[2], Dominik Haenni[1,3], Adam Pearson[1], Susan Ghazi[1], Milica Bugarski[1], Marcello Polesel[1], Michael Duss[4], Ehud M Landau[4], Andres Kaech[3], Urs Ziegler[3], Anne KM Lundby[5], Carsten Lundby[5], Petra S Dittrich[2], Andrew M Hall*[1,6].

[1]Institute of Anatomy, University of Zurich, Switzerland. [2]Department of Biosystems Science and Engineering, ETH Zurich, Switzerland. [3]Center for Microscopy and Image Analysis, University of Zurich, Switzerland. [4]Department of Chemistry, University of Zurich, Switzerland. [5]Institute of Physiology, University of Zurich, Switzerland, [6]Department of Nephrology, University Hospital Zurich, Switzerland.

Correspondence to: andrew.hall@uzh.ch

This file includes:

Captions for Supplementary Movies 1 to 3

Supplementary Movie 1. Mobility of swollen GFP-labeled mitochondria (green) along microtubules (labeled with SiR tubulin, red) is reduced in cells after treatment for 40 minutes with DFX, in comparison to control cells (acquisition rate = 1 frame/5 seconds, scale = 10 µm).

Supplementary Movie 2. Co-imaging of mitochondrial morphology (with GFP labeling, green) and energization state (with TMRM, red) reveals that mitochondrial swelling induced by DFX reverses upon addition of Iron (III) (200 µM) (acquisition rate = 1 frame/10 seconds, scale = 10 µm).

Supplementary Movie 3. Giant IMM vesicles prepared in PBS containing 0.8 mg/ml pHrodo were trapped in a microfluidic chamber and incubated with PBS containing 0.8 mg/ml pHrodo and 1 mM DFX. DFX reaches the membrane at 35 seconds. Note the rise in intensity of membrane-bound pHrodo (red) and the vesicle deformation at the end. Scale = 10 µm.
